# The Interplay of Rogue and Clustered Ryanodine Receptors Regulates Ca^2+^ Waves in Cardiac Myocytes

**DOI:** 10.3389/fphys.2018.00393

**Published:** 2018-04-26

**Authors:** Xudong Chen, Yundi Feng, Yunlong Huo, Wenchang Tan

**Affiliations:** ^1^State Key Laboratory of Turbulence and Complex Systems and Department of Mechanics and Engineering Science, College of Engineering, Peking University, Beijing, China; ^2^PKU-HKUST Shenzhen-Hong Kong Institution, Shenzhen, China; ^3^Shenzhen Graduate School, Peking University, Shenzhen, China

**Keywords:** Ca^2+^ wave, Ca^2+^ quark, anomalous subdiffusion, rogue ryanodine receptors, clustered ryanodine receptors

## Abstract

Ca^2+^ waves in cardiac myocytes can lead to arrhythmias owing to delayed after-depolarisations. Based on Ca^2+^ regulation from the junctional sarcoplasmic reticulum (JSR), a mathematical model was developed to investigate the interplay of clustered and rogue RyRs on Ca^2+^ waves. The model successfully reproduces Ca^2+^ waves in cardiac myocytes, which are in agreement with experimental results. A new wave propagation mode of “spark-diffusion-quark-spark” is put forward. It is found that rogue RyRs greatly increase the initiation of Ca^2+^ sparks, further contribute to the formation and propagation of Ca^2+^ waves when the free Ca^2+^ concentration in JSR lumen ([Ca^2+^]_lumen_) is higher than a threshold value of 0.7 mM. Computational results show an exponential increase in the velocity of Ca^2+^ waves with [Ca^2+^]_lumen_. In addition, more CRUs of rogue RyRs and Ca^2+^ release from rogue RyRs result in higher velocity and amplitude of Ca^2+^ waves. Distance between CRUs significantly affects the velocity of Ca^2+^ waves, but not the amplitude. This work could improve understanding the mechanism of Ca^2+^ waves in cardiac myocytes.

## Introduction

Ca^2+^ sparks due to the opening of clustered RyRs are the elementary Ca^2+^ release events in normal cardiac myocytes (Cheng et al., 1993; Cheng and Lederer, 2008), which could occur in self-propagating succession along the length, and contribute to waves of elevated Ca^2+^ concentration under some pathological conditions (López-López et al., 1995). Ca^2+^ waves have been observed in a diversity of cells (Ridgway et al., 1977; Fabiato, 1983; Cornellbell and Finkbeiner, 1991) and studied experimentally and theoretically (Fabiato and Fabiato, 1972; Fabiato, 1985; Backx et al., 1989; Swietach et al., 2010). Generating Ca^2+^ waves in myocytes is associated with RyRs gating and sarcoplasmic reticulum Ca^2+^ overload (Petrovic et al., 2015; Williams et al., 2017). Quarky Ca^2+^ release (QCR or Ca^2+^ quark) with a small amplitude and a long duration arising from rogue RyRs is another significant Ca^2+^ release mechanism (Wang et al., 2001; Cheng and Wang, 2002; Brochet et al., 2011; Shang et al., 2014). Hence, Ca^2+^ waves are a natural consequence of regenerative Ca^2+^ releases of both Ca^2+^ sparks and quarks. There is, however, lack of studies to relate Ca^2+^ waves to the interplay of Ca^2+^ sparks and quarks from the junctional sarcoplasmic reticulum (JSR). Based on the Fickian diffusion of cytoplasmic Ca^2+^, a computational model was developed to show the effects of rogue RyRs on Ca^2+^ waves under heart failure (Lu et al., 2010). Given the spark-width paradox from the Fickian diffusion models (Walker et al., 2014), the anomalous diffusion model can solve the problem and look more deeply into the mechanism of diffusion (Sato and Bers, 2011).

On the other hand, one of the challenges in developing models for Ca^2+^ waves is the inconsistence between computational and experimental free Ca^2+^ concentration in the cytoplasm ([Ca^2+^]_cyto_) (Izu et al., 2013). The computational results of [Ca^2+^]_cyto_ were ~20 μM (Chen et al., 2013) under physiological conditions or even as high as ~100 μM (Izu et al., 2001; Chen et al., 2014) under pathological conditions, which disagrees with the measured [Ca^2+^]_cyto_ of ~1 μM (Williams et al., 1985; Takamatsu and Wier, 1990). Although a “wave front sensitization” model showed [Ca^2+^]_cyto_ of ~1 μM (Keller et al., 2007), Sobie et al. indicated that elevated JSR Ca^2+^ level is a critical factor to raise RyRs open probability (Sobie et al., 2017). Hence, JSR Ca^2+^ regulation should be incorporated into the computational models of Ca^2+^ waves to show the decrease of Ca^2+^ flux through rogue and clustered RyRs as the JSR depletes (Sobie et al., 2004; Picht et al., 2011; Izu et al., 2013).

The objective of the study is to quantify the interplay of rogue and clustered RyRs on regulating Ca^2+^ waves in cardiac myocytes. A two-dimensional (2D) model of Ca^2+^ waves in the cytoplasm was proposed with considering the distribution of clustered and rogue RyRs on the JSR membrane. The anomalous subdiffusion of Ca^2+^ in the cytoplasm and JSR Ca^2+^ regulation were also included. The stochastic opening Ca^2+^ release units (CRUs) of rogue and clustered RyRs was regulated by free Ca^2+^ concentrations in both cytoplasm and JSR lumen. With these features, we showed the importance of rogue RyRs on the initiation and propagation of Ca^2+^ waves.

## Materials and methods

### Geometrical model

**Figure 1:**
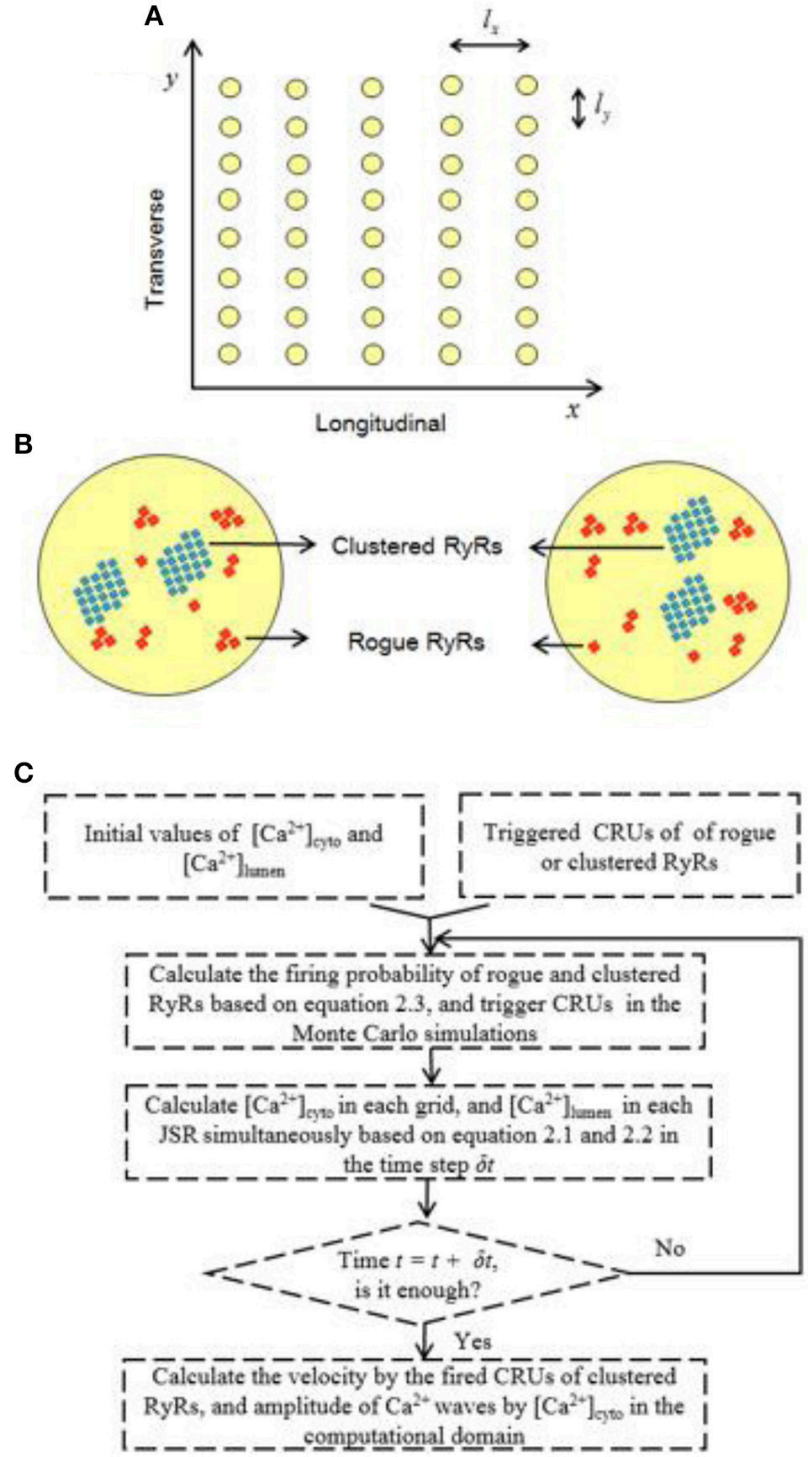
Schematic illustration of the 2D model for Ca^2+^ waves. **(A)** Schematic representative of the 2D geometrical model of a cardiac myocyte. The yellow circles denote JSRs. The spacing intervals between JSRs: *l*_*x*_ = 2 μm and *l*_*y*_ = 0.8 μm. **(B)** Schematic representative of two JSRs, which include randomly distributed clustered and rogue RyRs (*l* = 0.1 μm). **(C)** A flow diagram for the Monte Carlo simulations.

Considering the quasi-isotropic diffusion of Ca^2+^ in the cytoplasm (Izu et al., 2001), we adopted a 2D model to mimic Ca^2+^ waves. Figure 1A shows the geometrical model of a cardiac myocyte, the x- and y-directions of which refer to the longitudinal axis and z-line, respectively. Baddeley et al. have experimentally observed the RyR distribution on the JSR membrane (Baddeley et al., 2010). Most RyR channels form regular arrays, defined as “clustered RyRs.” Others are rogue RyRs uncoupled from the clustered RyRs. Clustered and rogue RyRs are randomly distributed. Figure 1B shows schematic representative of the CRU distribution on JSRs, which includes CRUs of clustered RyRs (~22 RyR channels in a CRU) and CRUs of rogue RyRs (~3 RyR channels in a CRU). CRUs of clustered RyRs (~2 CRUs in a JSR) are surrounded by randomly distributed CRUs of rogue RyRs (~8 CRUs in a JSR).

### Governing equations

Ca^2+^ release events are simulated synchronously by a hybrid model, which consists of two parts: a model of Ca^2+^ waves in the cytoplasm and a model of Ca^2+^ blinks in JSRs. The reaction-diffusion system for Ca^2+^ waves in the cytoplasm based on the anomalous subdiffusion model, including the distribution of clustered and rogue RyRs, is described as follows:

(1) $$\begin{array}{l} \frac{\partial {\left[{\text{Ca}}^{2+}\right]}_{\text{cyto}}}{\partial t}=D_{x}\frac{\partial^{\beta }{\left[{\text{Ca}}^{2+}\right]}_{\text{cyto}}}{\partial x^{\beta }}+D_{y}\frac{\partial^{\beta }{\left[{\text{Ca}}^{2+}\right]}_{\text{cyto}}}{\partial y^{\beta }}+J_{\text{dye}}\\ \;+J_{\text{buffer-cyto}}+J_{\text{pump}}+J_{\text{clustered}}+J_{\text{rogue}}, \end{array}$$

where [Ca^2+^]_cyto_ is the free Ca^2+^ concentration in the cytoplasm, *t* is time, *x* and *y* are the spatial coordinates, *D*_*x*_(= 300 μm^2^s^−1^) and *D*_*y*_ (= 150 μm^2^s^−1^) denote the Ca^2+^ diffusion coefficients for anisotropic diffusion. The anomalous subdiffusion order β is 2.25. *J*_dye_ is the flux due to the Ca^2+^ fluorescent indicator dye, Fluo-4-AM, in the cytoplasm. *J*_buffer−cyto_ is the flux due to the endogenous stationary buffers. *J*_pump_ is the pumping rate of SR Ca^2+^-ATPase. SR pumps will be started when ${\left[{\text{Ca}}^{2+}\right]}_{\text{cyto}}$ exceeds the resting Ca^2+^ concentration level (0.1 μM). The detailed description is in the Appendix A in Supplementary Material. On the other hand, the balance equation for Ca^2+^ blinks in each JSR is written as:

(2) $$\frac{\partial {\left[{\text{Ca}}^{2+}\right]}_{\text{lumen}}}{\partial t}=J_{\text{release}-\text{lumen}}+J_{\text{buffer}-\text{lumen}}+J_{\text{refill}},$$

where [Ca^2+^]_lumen_ is the free Ca^2+^ concentration in the lumen of a JSR. *J*_release−lumen_ denotes the Ca^2+^ release flux caused by opening of clustered RyRs (*J*_clustered_) and rogue RyRs (*J*_rogue_). *J*_buffer−lumen_ is the Ca^2+^ flux due to the buffer, calsequestrin, in the JSR. *J*_refill_ is the refilled Ca^2+^ flux to the JSR. The detailed description is in the Appendix B in Supplementary Material. Various parameters of the dye and buffers in the cytoplasm and JSR lumen are listed in Table 1, similar to previous studies (Chen et al., 2013; Kong et al., 2013).

**Table 1:** Standard parameter values for the dye and buffers.

**Dye or buffers**	**[F]_T_ or [B_n_]_T_ (μM)**	**$k_{F}^{+}$or $k_{n}^{+}$ (μM^−1^s^−1^)**	**$k_{F}^{-}$or$k_{n}^{-}$ (s^−1^)**
**PARAMETERS IN CYTOPLASM**			
Fluo-4-AM	50	80	90
Calmodulin	24	100	38
Troponin	70	39	20
SR	47	115	100
SL	1,124	115	1,000
**PARAMETERS IN JSR LUMEN**			
Calsequestrin	14,000	100	60,000

### Firing probability of rogue and clustered RyRs

The firing probability per unit time for CRUs of rogue or clustered RyRs is determined by Ca^2+^ concentrations in the cytoplasm and JSR (Györke and Gyorke, 1998; Qin et al., 2008, 2009), which can be expressed as:

(3) $$P_{\text{firing}}=P_{\text{cyto}}\cdot \Phi_{\text{lumen}},$$

where *P*_cyto_ and Φ_lumen_ refer to the firing probability per unit time of Ca^2+^ release events controlled by ${\left[\text{C}{\text{a}}^{2+}\right]}_{\text{cyto}}$ and [Ca^2+^]_lumen_, respectively. The detailed description is in the Appendix C in Supplementary Material.

### Numerical solutions

The 2D computational domain of a cardiac cytoplasm (20 × 20 μm^2^) was meshed with squares of 0.1 × 0.1 μm to simulate Ca^2+^ release events from multiple JSRs. JSRs (i.e., yellow circles with radius of 0.3 μm in Figure 1A) are uniformly distributed in the computational domain with *l*_*x*_ (2 μm) along x-axis and *l*_*y*_ (0.8 μm) along y-axis. Moreover, CRUs are stochastically distributed at nodes within each JSR, which includes 8 CRUs of rogue RyRs (*N*_rogue_ = 8) and 2 CRUs of clustered RyRs (*N*_clustered_ = 2). As shown in Figure 1C, Equations (1–3) were solved using a FORTRAN-developed program similar to a recent study (Chen et al., 2018). The shifted Grünwald formula of center difference (Tadjeran and Meerschaert, 2007) was used to discretize the fractional differential term in Equation (1) as:

(4) $$\frac{\partial^{\alpha }{\left[{\text{Ca}}^{2+}\right]}_{\text{cyto}}(x,y,t)}{\partial x^{\alpha }}\;=\;\frac{1}{h^{\alpha }}\underset{M\to \infty }{\lim}\sum_{k\;=\;0}^{M}g_{k}{\left[{\text{Ca}}^{2+}\right]}_{\text{cyto}}(x-(k-1)h,y,t)$$

(5) $$\frac{\partial^{\alpha }{\left[{\text{Ca}}^{2+}\right]}_{\text{cyto}}(x,y,t)}{\partial y^{\alpha }}=\frac{1}{h^{\alpha }}\underset{M\to \infty }{\lim}\sum_{k\;=\;0}^{M}g_{k}{\left[{\text{Ca}}^{2+}\right]}_{\text{cyto}}(x,y-(k-1)h,t),$$

where $g_{k}=\frac{\Gamma \left(k-\alpha \right)}{\Gamma \left(k+1\right)}$, Γ denotes the Gamma function. α = β−1 = 1.25, *k* is an integer with α < *k* < α+1, and *h* is the mesh size. Free Ca^2+^ concentrations in the cytoplasm and JSR were calculated simultaneously. The variable time step algorithm was used. The zero-flux boundary condition was set to the 2D computational domain of a cardiac cytoplasm for the Monte Carlo simulations.

## Results and discussion

### Interplay of rogue and clustered RyRs in neighbor JSRs

**Figure 2:**
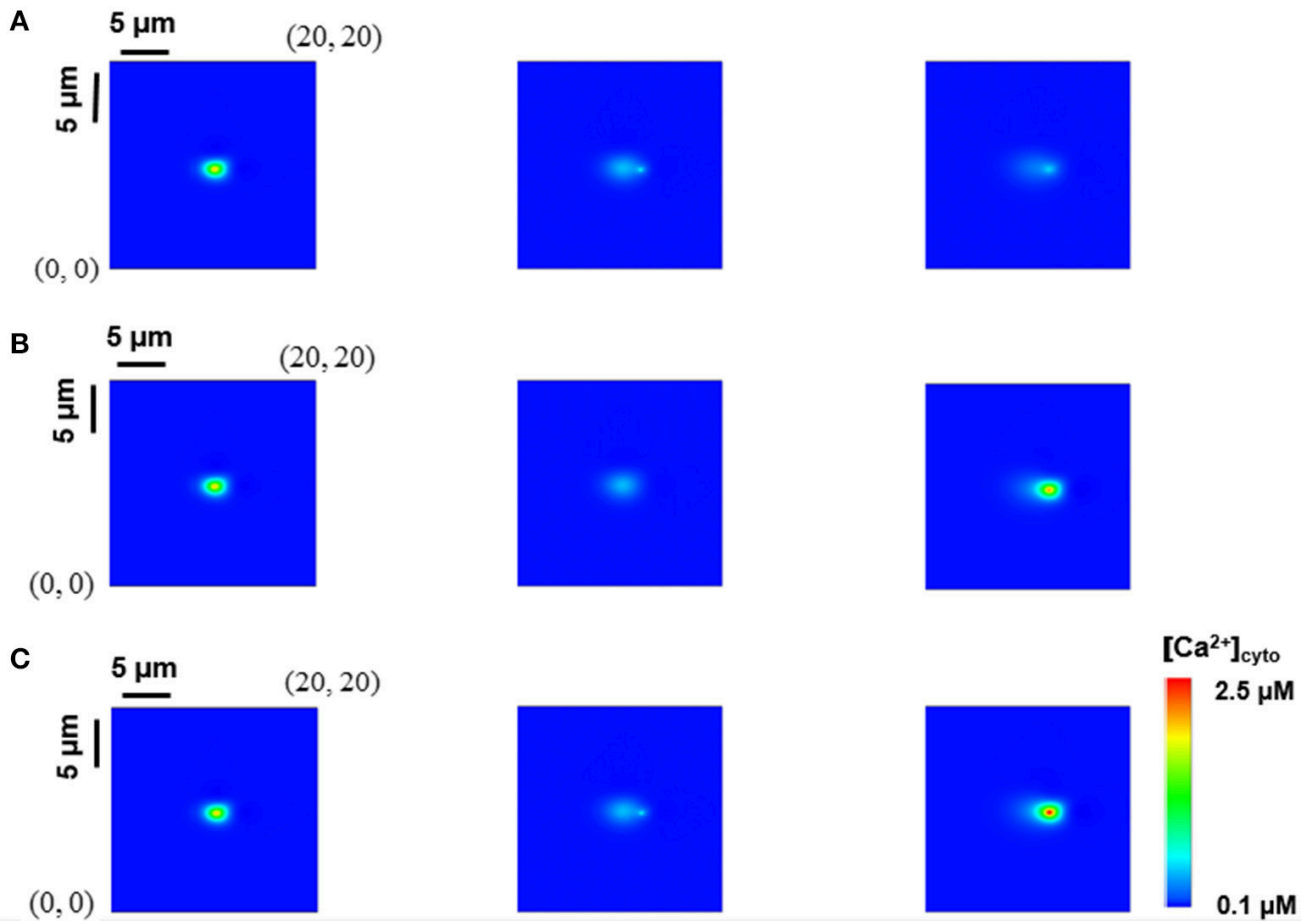
Interplay of rogue and clustered RyRs in neighbor JSRs. Snapshots of Ca^2+^ release events in a computational region of 20 × 20 μm^2^ were taken at 10, 20, and 40 ms from left to right when a CRU of clustered RyRs was fired. **(A)** Rogue RyRs in a neighbor JSR are activated. **(B)** Clustered RyRs in a neighbor JSR are initiated by a Ca^2+^ spark directly. **(C)** Clustered RyRs are triggered with the help of rogue RyRs.

We have recently studied the effects of rogue RyRs on single Ca^2+^ sparks and quarks using the model in Equations (1–5), which was validated against the experimental measurements in rat cardiac myocytes (Chen et al., 2018). Here, we used the experimentally-validated numerical model to investigate whether a Ca^2+^ spark could trigger rogue and clustered RyRs in neighbor JSRs or not. Snapshots of Ca^2+^ release events in a computational domain of 20 × 20 μm^2^ were taken at 10, 20, and 40 ms when a CRU of clustered RyRs was fired. Figures 2A,B show rogue RyRs at point (11.9, 9.6) and clustered RyRs at point (12.0, 9.6), respectively, activated by the Ca^2+^ spark at point (10.0, 9.6). Moreover, the release of clustered RyRs could trigger other CRUs of clustered RyRs in neighbor JSRs with the help of rogue RyRs, as shown in Figure 2C. The results demonstrate that Ca^2+^ sparks from the opening CRUs of clustered RyRs could activate CRUs of clustered RyRs and rogue RyRs in neighbor JSRs to increase [Ca^2+^]_cyto_.

### Initiation of Ca^2+^ waves

**Figure 3:**
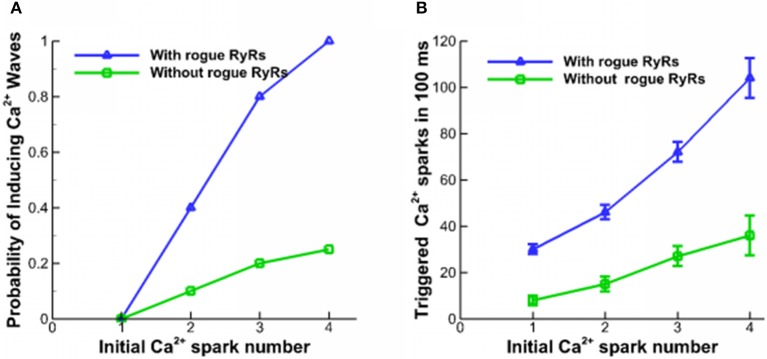
Properties of Ca^2+^ waves. **(A)** The probability of inducing Ca^2+^ waves triggered by different numbers of Ca^2+^ sparks. **(B)** The number of triggered Ca^2+^ sparks in 100 ms.

Ca^2+^ waves could be triggered in a domain where [Ca^2+^]_cyto_ is higher than the resting Ca^2+^ concentration level (Lu et al., 2010; Izu et al., 2013). A Ca^2+^ spark with a large current or several neighbor Ca^2+^ sparks could also trigger Ca^2+^ waves (Chen et al., 2014). Here, several neighbor Ca^2+^ sparks are used to activate Ca^2+^ waves in the simulation. The lowest propagation velocity of Ca^2+^ waves was detected in the range of 40–110 μm/s (Cheng et al., 1996). Since the disappearance of Ca^2+^ waves is related to a progressive decline of the wave propagation velocity, the longitudinal velocity is assumed to be higher than 40 μm/s. Figure 3A shows the probability of inducing Ca^2+^ waves as a function of the number of Ca^2+^ sparks initiating from a corner in a square of 20 × 20 μm^2^ for 100 ms. The beginning level of [Ca^2+^]_lumen_ is 1.0 mM. The initial Ca^2+^ sparks arise from one CRU of clustered RyRs. Since Ca^2+^ quarks increase Ca^2+^ concentration in the cytoplasm, they enhance the probability of inducing Ca^2+^ waves in myocytes. Accordingly, Figure 3B shows the number of triggered Ca^2+^ sparks in 100 ms. A single Ca^2+^ spark could not form Ca^2+^ waves while four neighbor Ca^2+^ sparks with the help of rogue RyRs guarantee the formation of Ca^2+^ waves.

### Propagation of Ca^2+^ waves

**Figure 4:**
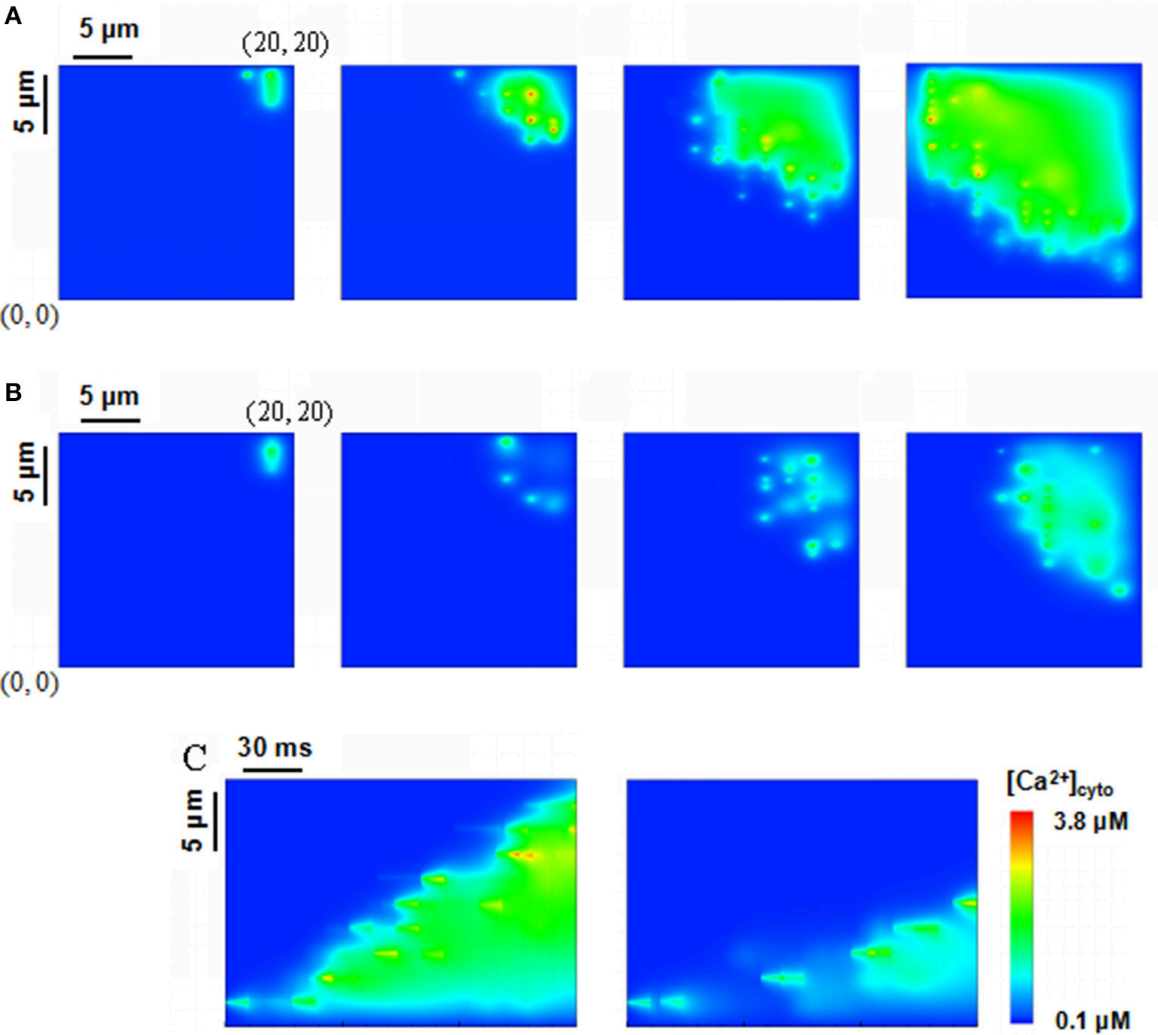
Ca^2+^ waves triggered by four sparks at the corner of a computational domain, i.e., (18, 19.2), (18, 18.4), (18, 17.6), (18, 16.8) in the region of 20 × 20 μm^2^. The beginning level of [Ca^2+^]_lumen_ is 1.0 mM. **(A,B)** Snapshots of Ca^2+^ waves initially triggered by Ca^2+^ sparks with rogue RyRs **(A)** and without considering rogue RyRs **(B)** at 10, 50, 100, 150 ms from left to right. **(C)** Computational results for line-scan images of [Ca^2+^]_cyto_ with and without rogue RyRs at the line of *y* = 16.8.

Ca^2+^ waves in Figure 4 were generated in a computational domain of 20 × 20 μm^2^ and recorded at 10, 50, 100, and 150 ms from left to right when the beginning level of [Ca^2+^]_lumen_ is 1.0 mM. Four Ca^2+^ sparks were initiated at points (18, 19.2), (18, 18.4), (18, 17.6), and (18, 16.8) with considering rogue RyRs. A comparison of Figures 4A,B indicates much faster Ca^2+^ waves and higher amplitude when the effects of rogue RyRs are included in the computational model. Moreover, we found the “spark-diffusion-quark-spark” mode. Ca^2+^ released from clustered RyRs diffuses to a neighbor JSR, rogue RyRs are firstly activated in a stochastic manner to form Ca^2+^ quarks, and subsequently they make activation of clustered RyRs to produce a Ca^2+^ spark. The CRUs on the next z-line repeat the process to release Ca^2+^ in the cytoplasm.

Figure 4C shows the computational results for line-scan images of Ca^2+^ concentration with and without considering rogue RyRs at the line of *y* = 16.8. The results reveal that rogue RyRs accelerate the propagation of waves by triggering more Ca^2+^ sparks. The longitudinal propagating velocity has mean ± *SD* value of 95.9 ± 8.0 μm/s, which agrees with the experimental records (typically 100 μm/s) (Takamatsu and Wier, 1990; Wier and Blatter, 1991). Furthermore, Ca^2+^ concentration in the simulation is in the range of 0.1–3.8 μM. The computational predictions are close to the experimental measurements (0.5–1.2 μM; Williams et al., 1985), and less than previous simulations (Izu et al., 2001; Chen et al., 2013).

### Importance of [Ca^2+^]_lumen_-dependent regulation

**Figure 5:**
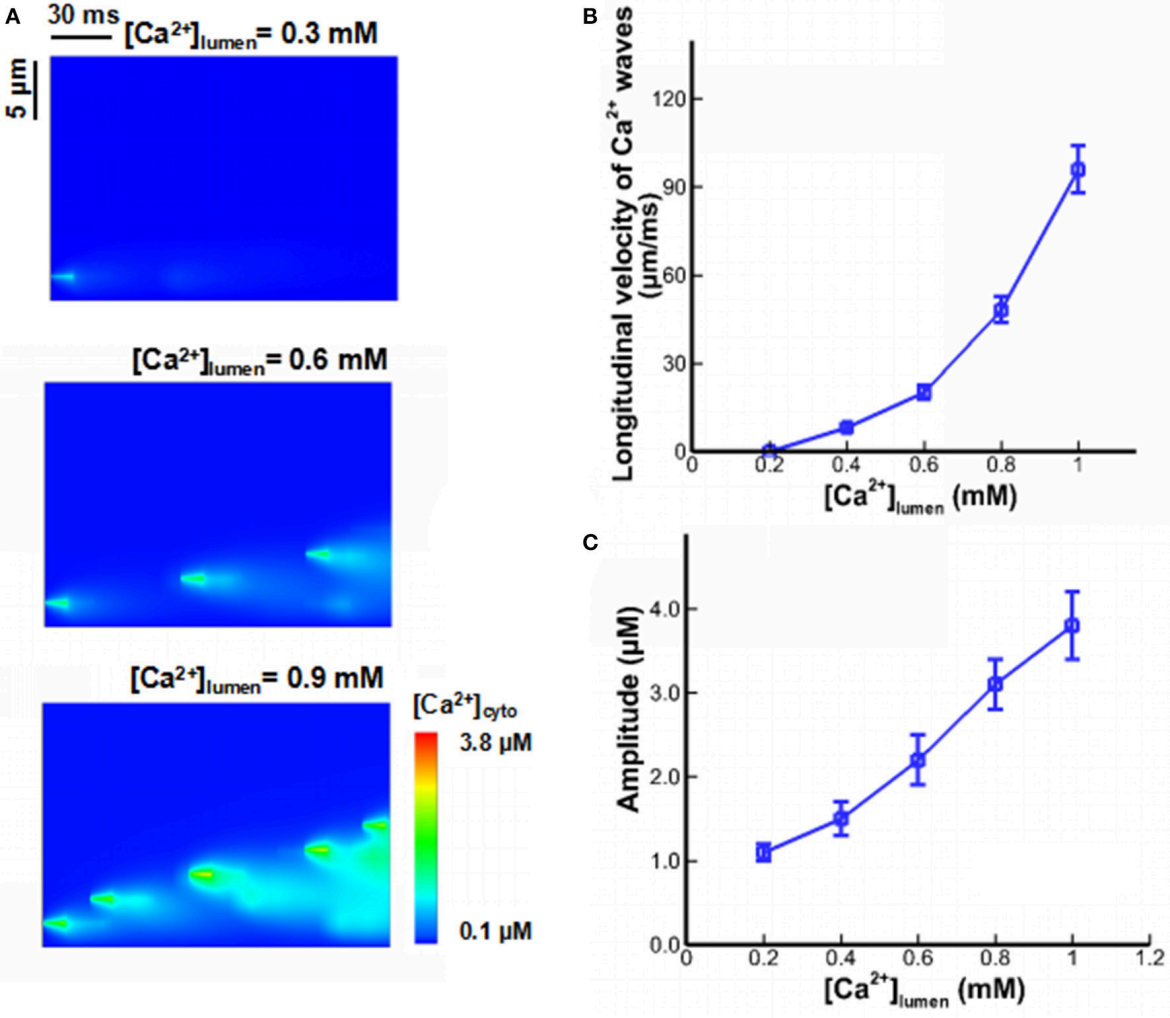
Ca^2+^ waves under different [Ca^2+^]_lumen._ **(A)** Computational results for line-scan images of [Ca^2+^]_cyto_ at the line of *y* = 16.8 when [Ca^2+^]_lumen_ = 0.3, 0.6, and 0.9 mM. **(B)** The longitudinal velocity of Ca^2+^ waves affected by [Ca^2+^]_lumen_. **(C)** The amplitude of Ca^2+^ waves affected by [Ca^2+^]_lumen_.

In previous mathematical models of Ca^2+^ waves, [Ca^2+^]_lumen_-dependent regulation was not considered, and Ca^2+^ fluxes from CRUs were related to the current and duration of Ca^2+^ sparks and quarks only. We determined the effects of [Ca^2+^]_lumen_-dependent regulation on properties of Ca^2+^ waves when the beginning level of [Ca^2+^]_lumen_ was set to 0.3, 0.6, and 0.9 mM. Local variation in the waves is shown in Figure 5A. Ca^2+^ waves are very sensitive to [Ca^2+^]_lumen_. There is an exponential increase in the velocity of Ca^2+^ waves with the increase of [Ca^2+^]_lumen_, as shown in Figure 5B. Moreover, a threshold value of [Ca^2+^]_lumen_ (i.e., >0.7 mM) exists for generation of steady Ca^2+^ waves. Figure 5C shows the amplitude of Ca^2+^ waves linearly increase with [Ca^2+^]_lumen_ because of the large driving force ([Ca^2+^]_lumen_-[Ca^2+^]_cyto_) of Ca^2+^ sparks and quarks.

### Effects of parameters of rogue RyRs

**Figure 6:**
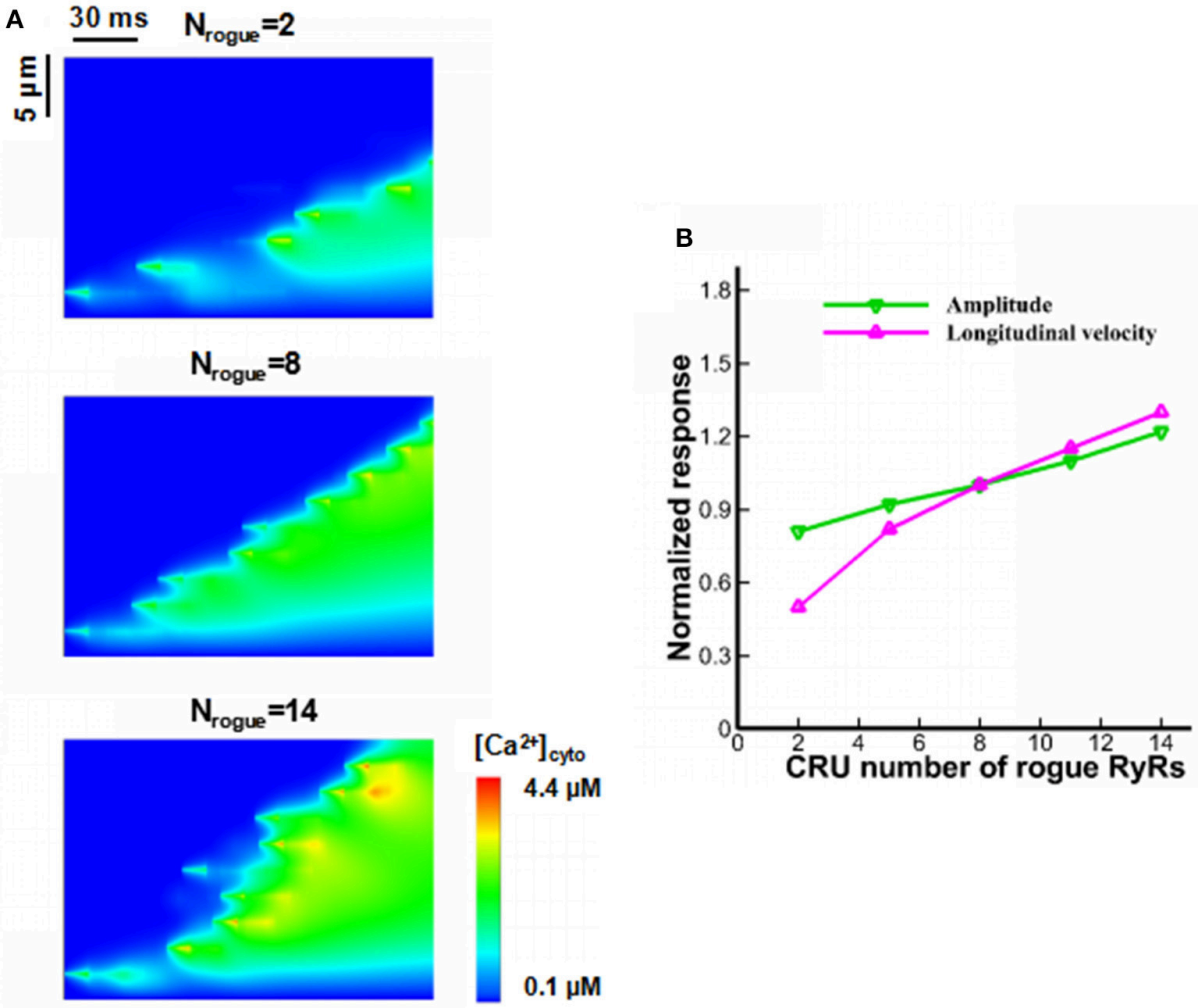
Effects of CRU number of rogue RyRs in a JSR on wave propagation. **(A)** Computational results for line-scan images of [Ca^2+^]_cyto_ at the line of *y* = 16.8 when CRU number of rogue RyRs has values of 2, 8, and 14. **(B)** The amplitude and longitudinal velocity of Ca^2+^ waves when N_rogue_ varies from 2 to 14.

There are a large number of randomly-distributed rogue RyRs near clustered RyRs. Sensitivity analysis on Ca^2+^ waves was performed with respect to the changes in CRU number of rogue RyRs in a JSR (N_rogue_). Figure 6A shows computational results for line-scan images of [Ca^2+^]_cyto_ at the line of *y* = 16.8 when CRU number of rogue RyRs in a JSR are 2, 8, and 14. As shown in Figure 6B, the amplitude and velocity increase with the increased CRU number of rogue RyRs because of high Ca^2+^ quarks and Ca^2+^ sparks.

The Ca^2+^ release per CRU of rogue RyRs is mainly determined by the current through rogue RyRs (*I*_rogue_) and the duration of current flow (*T*_rogue_). The current and duration of rogue RyRs are 0.15 pA and 20 ms in Figures 2–6. Table 2 presents properties of Ca^2+^ waves for different values of (*I*_rogue_ × *T*_rogue_). There is a strong correlation between Ca^2+^ release through rogue RyRs and wave properties. When the release time of rogue RyRs decreases by half to 10 ms with the current of 0.15 pA, the longitudinal velocity and amplitude of waves decrease. When the current increases from 0.15 to 0.3 pA with the duration of 20 ms, Ca^2+^ waves have higher values of amplitude and longitudinal velocity. Hence, the release amount of rogue RyRs characterized by (*I*_rogue_ × *T*_rogue_) is a significant parameter to affect wave properties.

**Table 2:** Effects of Ca^2+^ release per CRU of rogue RyRs on properties of Ca^2+^ waves.

***I*_rogue_ × *T*_rogue_**	**0.15 × 10**	**0.15 × 20**	**0.3 × 20**
Longitudinal velocity (μm/s)	76.2	95.9	154.0
SEM	6.4	8.0	14.4
Amplitude (μM)	3.4	3.8	4.5
SEM	0.3	0.3	0.4

**Figure 7:**
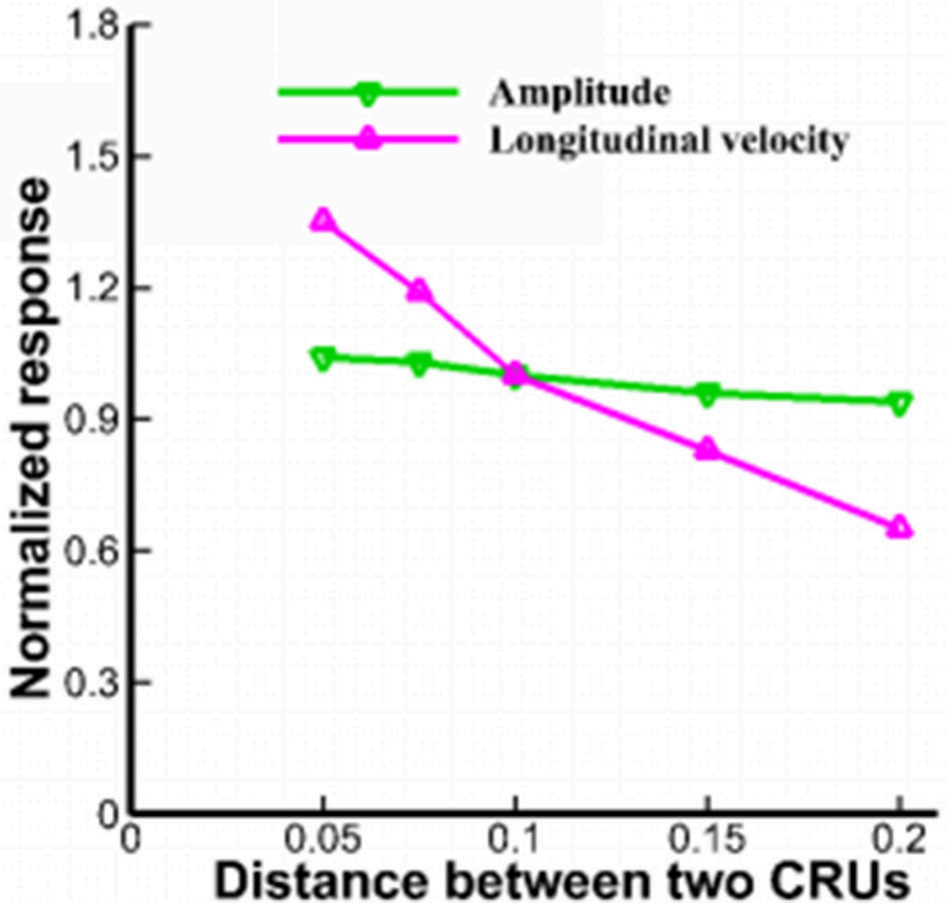
The amplitude and longitudinal velocity of Ca^2+^ waves when *l* varies from 0.05 to 0.2 μm.

Lu et al. pointed out that rogue RyRs were scattered over the 2D plane randomly (Lu et al., 2010). However, fluorescent imaging showed that QCR events were detected almost at the same site as Ca^2+^ sparks (Brochet et al., 2011). The stochastic gating of a cluster contains 10–100 RyRs in a JSR and the mean number of RyRs is ~21.6 (Soeller et al., 2007; Baddeley et al., 2009). Figure 7 shows that the distance between CRUs in the range of 0.05–0.2 μm has slight effects on the amplitude of Ca^2+^ waves. However, the longitudinal velocities are 144.3 ± 13.1 and 63.6 ± 7.8 μm/s, respectively with respect to the distance of 0.05 and 0.2 μm. Therefore, the distance between CRUs of rogue RyRs and clustered RyRs should be taken into consideration in the propagation of Ca^2+^ waves.

### Effects on activities from subcellular to cellar levels

When a myocyte is under paced, several spontaneous Ca^2+^ sparks or quarks can occur in the cytoplasm. It is, however, difficult to induce Ca^2+^ waves that require abundant currents to trigger cardiac action potentials (Izu et al., 2013). In some pathological conditions (e.g., arrhythmias), Ca^2+^ waves occur in cardiac myocytes and affect the heart's normal function (Lakatta and Guarnieri, 1993). Ten Tusscher and Panfilov developed a ventricular cell model including subspace calcium dynamics that controls L-type calcium current and calcium-induced calcium release (CICR) (ten Tusscher and Panfilov, 2006). The model was used to study the effects of *I*_*Na*_ recovery dynamics in combination with action potential duration (ADP) restitution on alternans and spiral breakup. On the other hand, the present results suggest that Ca^2+^ release from JSRs is prone to be initiated to cause Ca^2+^ waves with the help of rogue RyRs when [Ca^2+^]_lumen_ is large enough. Moreover, when the CRU number of rogue RyRs in a JSR or Ca^2+^ release per CRU of rogue RyRs is large enough, or the distance between CRUs is small, the membrane potential would be elevated significantly to trigger action potential in single myocytes.

### Potential implications

This study shows some implications to heart diseases relevant to Ca^2+^ waves in cardiac myocytes. According to our simulations, JSR Ca^2+^ overload could increase RyR opening probability and generate Ca^2+^ waves in heart (Williams et al., 2017). Mutation in RyRs could trigger ventricular tachycardia and sudden cardiac death (Lehnart et al., 2006). Moreover, the amplitude and velocity of Ca^2+^ waves are significantly affected by the parameters of rogue RyRs, which may contribute to the formation of fibrillation (Macquaide et al., 2015) and arrhythmias (Ter Keurs and Boyden, 2007). A reduction of CRU number, the averaged current and releasing time of rogue RyRs resulted in an inhibition of Ca^2+^ waves or dyssynchronous Ca^2+^ transients in myocytes of congestive heart failure (Louch et al., 2013). Therefore, inhibition of Ca^2+^ quarks through rogue RyRs may be a promising therapeutic target to prevent fibrillation in congestive heart failure.

### Critique of the study

The present study showed Ca^2+^ waves relevant to the interplay of rogue and clustered RyRs. It addressed the importance of rogue RyRs to increase the initiation of Ca^2+^ sparks, the incidence and propagation of Ca^2+^ waves when [Ca^2+^]_lumen_ is large. The amplitude and velocity of Ca^2+^ waves are in agreement with the experimental measurements. However, the model should be improved such that more parameters are added to investigate the mechanisms of Ca^2+^ waves. This study used the fixed release time of rogue and clustered RyRs. It should be a random variable depending on JSR regulation. The detailed structure of clustered RyRs should be taken into consideration because it can influence the frequency of Ca^2+^ sparks (Walker et al., 2014). Moreover, the present study comes from the assumption of a 2D model in healthy myocytes. A 3D model should be developed to investigate Ca^2+^ waves in future studies.

## Conclusion

We developed a mathematical model to investigate the interplay of rogue and clustered RyRs on regulating Ca^2+^ waves in the cytoplasm. The computational results on Ca^2+^ waves agree with experimental measurements in cardiac myocytes. It shows that four neighbor Ca^2+^ sparks at the corner of a cardiac myocyte could induce Ca^2+^ waves. Ca^2+^ quarks increase the probabilities of triggering Ca^2+^ sparks and speed up the propagation of Ca^2+^ waves at high [Ca^2+^]_lumen_. A new wave propagation mode of “spark-diffusion-quark-spark” is put forward. Particularly, Ca^2+^ waves could occur only when [Ca^2+^]_lumen_ is higher than a threshold value of 0.7 mM. More rogue RyRs in a JSR result in more opening CRUs of rogue and clustered RyRs. Besides, Ca^2+^ release from CRUs of rogue RyRs is a strong factor of wave properties. The velocity of Ca^2+^ waves is affected significantly by the distance between CRUs. This study helps to understand basic mechanisms of Ca^2+^ waves in cardiac myocytes.

## Author contributions

XC, YH, and WT designed and performed the numerical calculation; YF analyzed and interpreted data; XC, YH, and WT wrote the manuscript.

### Conflict of interest statement

The authors declare that the research was conducted in the absence of any commercial or financial relationships that could be construed as a potential conflict of interest.
