# Supplementary material for: The Interplay of Rogue and Clustered Ryanodine Receptors Regulates Ca2+ Waves in Cardiac Myocytes
Source: Front Physiol. 2018 Apr 26;9:393. doi: 10.3389/fphys.2018.00393 (PMC5932313; doi:10.3389/fphys.2018.00393)
Supplement: Supplementary file 1 [file Data_Sheet_1.docx]

**APPENDIX A**

Anomalous space subdiffusion in the spatial reaction-diffusion model is defined though the relations (Metzler and Klafter, 2000)

$\frac{\partial^{2.25}[\text{Ca}^{2+}]_{\text{cyto}}(x,y,t)}{\partial x^{2.25}}=\frac{1}{\Gamma(0.75)}\frac{\partial^{3}}{\partial x^{3}}\int_{0}^{x} \frac{[\text{Ca}^{2+}]_{\text{cyto}}(\tau,y,t)}{(x-\tau)^{0.25}}d\tau\text{，}$ (A1)

$\frac{\partial^{2.25}[\text{Ca}^{2+}]_{\text{cyto}}(x,y,t)}{\partial y^{2.25}}=\frac{1}{\Gamma(0.75)}\frac{\partial^{3}}{\partial y^{3}}\int_{0}^{y} \frac{[\text{Ca}^{2+}]_{\text{cyto}}(x,\tau,t)}{(y-\tau)^{0.25}}d\tau\text{，}$ (A2)

where $\Gamma$ denotes the Gamma function.

Ca^2+^ fluxes in Equation (1) can all be defined as:

$J_{\text{dye-cyto}}=-k_{\text{F}}^{+}[\text{Ca}^{\text{2+}}]_{\text{cyto}}([\text{F}]_{\text{T}}-[\text{CaF}])+k_{\text{F}}^{-}[\text{CaF}],$ (A3)

$J_{\text{buffer-cyto}}=\sum_{\text{n}} -\frac{\partial[\text{CaB}_{\text{n}}]}{\partial t},$ (A4)

$\frac{\partial[\text{CaB}_{\text{n}}]}{\partial t}=k_{\text{n}}^{+}[\text{Ca}^{\text{2+}}]_{\text{cyto}}([\text{B}_{\text{n}}]_{\text{T}}-[\text{CaB}_{\text{n}}])-k_{\text{n}}^{-}[\text{CaB}_{\text{n}}],$ (A5)

$J_{\text{pump}}=-\frac{V_{\text{pump}}^{\max}([\text{Ca}^{\text{2+}}]_{\text{cyto}})^{\text{h}}}{(K_{\text{pump}})^{\text{h}}+([\text{Ca}^{\text{2+}}]_{\text{cyto}})^{\text{h}}},$ (A6)

where the subscript ‘n’ refers to each buffer in the cytoplasm, the superscript ‘h’ refers to the Hill constant; $[\text{F}]_{\text{T}}$ and $[\text{B}_{\text{n}}]_{\text{T}}$ represent the total concentrations of the indicator and buffers, respectively. $[\text{CaF}]$ and $[\text{CaB}_{\text{n}}]$ are the concentrations of Ca^2+^-bound complexes; $k_{\text{F}}^{+}$，$k_{\text{F}}^{-}$，$k_{\text{n}}^{+}$ and $k_{\text{n}}^{-}$ are the reaction kinetic parameters. $K_{\text{pump}}$ is the affinity constant, and $V_{\text{pump}}^{\max}$ is the maximum rate for SR pumps. Values of the parameters are based on a previous study (Chen et al., 2013). Moreover, $J_{\text{rogue}}$ and $J_{\text{clustered}}$ are the Ca^2+^ fluxes released by the CRUs of rogue and clustered RyRs respectively, which can be written as:

$J_{\text{rogue}}=\sigma_{\text{rogue}}\sum_{i,j} \delta(x-x_{i}^{\text{rogue}},y-y_{j}^{\text{rogue}})S(x_{i}^{\text{rogue}},y_{j}^{\text{rogue}},t;T_{\text{rogue}}),$ (A7)

$J_{\text{clustered}}=\sigma_{\text{clustered}}\sum_{i,j} \delta(x-x_{i}^{\text{clustered}},y-y_{j}^{\text{clustered}})S(x_{i}^{\text{clustered}},y_{j}^{\text{clustered}},t;T_{\text{clustered}}),$ (A8)

where $\delta$ is the Dirac delta function and $S$ is a stochastic function for the opening of CRUs, $(x_{i}^{\text{rogue}},\text{ }y_{j}^{\text{rogue}})$ and $(x_{i}^{\text{clustered}},\text{ }y_{j}^{\text{clustered}})$are the positions of CRUs of rogue RyRs and clustered RyRs in the 2D plane respectively. The release time for rogue and clustered RyRs are defined as $T_{\text{rogue}}$= 20 ms (Brochet et al., 2011) and $T_{\text{clustered}}$= 10 ms (Smith et al., 1998). The equivalent source strength of rogue RyRs and clustered RyRs (Li et al., 2017), which are used to adjust the Ca^2+^ molar flux of the line source to a point source, are expressed by

$\sigma_{\text{rogue}}=0.64I_{\text{rogue}}([\text{Ca}^{\text{2+}}]_{\text{lumen}}-[\text{Ca}^{\text{2+}}]_{\text{cyto}})/2F,$ (A9)

$\sigma_{\text{clustered}}=0.64I_{\text{clustered}}([\text{Ca}^{\text{2+}}]_{\text{lumen}}-[\text{Ca}^{\text{2+}}]_{\text{cyto}})/2F,$ (A10)

where the Faraday constant $F$ is 96500 C·mol^-1^. $I_{\text{rogue}}$ and $I_{\text{clustered}}$ are the average currents through rogue and clustered RyRs. In general, currents for modeling Ca^2+^ waves are larger than that under normal physiological conditions (Izu et al., 2001; Chen et al., 2013). So $I_{\text{rogue}}$ and $I_{\text{clustered}}$ are set to be 0.15 pA/mM and 1.5 pA/mM, about twice normal physiological currents of CRUs (0.07 pA/mM and 0.7 pA/mM (Li et al., 2017)). The value 0.64 in Equations (A9) and (A10) is the conversion factor, which is used to give the identical Ca^2+^ distribution in 2D because Ca^2+^ are released from the JSR lumen into a 3D volume (Izu et al., 2001).

**APPENDIX B**

Decreased Ca^2+^ release flux caused by the opening of CRUs in a JSR is denoted by

$J_{\text{release-lumen}}\text{=}-J_{\text{rogue}}-J_{\text{clustered}}$. (B1)

The Ca^2+^ flux due to the buffer in the JSR lumen is

$J_{\text{buffer-lumen}}\text{=}-k_{\text{cal}}^{+}[\text{Ca}^{\text{2+}}]_{\text{lumen}}([\text{B}_{\text{cal}}]_{\text{T}}-[\text{CaB}_{\text{cal}}])\text{+}k_{\text{cal}}^{-}[\text{CaB}_{\text{cal}}],$ (B2)

where $[\text{B}_{\text{cal}}]_{\text{T}}$ represents the total concentration of calsequestrin. $[\text{CaB}_{\text{cal}}]$ is the concentration of Ca^2+^-bound complexes in the JSR; $k_{\text{cal}}^{+}$ and $k_{\text{cal}}^{-}$ are the reaction kinetic parameters of calsequestrin. These parameters are listed in Table 1.

The refilled Ca^2+^ flux $J_{\text{refill}}$ is expressed by

$J_{\text{refill}}=([\text{Ca}^{\text{2+}}]_{\text{NSR}}-[\text{Ca}^{\text{2+}}]_{\text{lumen}})/\tau_{\text{refill}},$ (B3)

where free Ca^2+^ concentration in network sarcoplasmic reticulum (NSR) [Ca^2+^]_NSR_ is 1.0 mM, time constant for Ca^2+^ transfer between JSR and NSR $\tau_{\text{refill}}$ is 10 ms, and the volume of a JSR lumen is 1×10^-11^ μL (Sobie et al., 2004). The beginning level of [Ca^2+^]_lumen_ is 1.0 mM.

**APPENDIX C**

According to the coupled RyR gating model (Zahradnikova et al., 2010), $P_{\text{cyto}}$ can be expressed as:

$P_{\text{cyto}}=1-(1-P{}_{\text{RyR}})^{n{}_{\text{RyR}}},$ (C1)

where $P_{\text{RyR}}$, a function of [Ca^2+^]_cyto_, is the firing probability per unit time of a single RyR channel. $n_{\text{RyR}}$ is the RyR number in a CRU for rogue ($n_{\text{RyR}}$= 3) or clustered ($n_{\text{RyR}}$= 22) RyRs. Here, $\Phi_{\text{lumen}}$ is written as:

(C2)

where $\varphi$ is an empirical power function corresponded to [Ca^2+^]_lumen_ and given in Walker et al.’s model (Walker et al., 2014), *m* is the regulation coefficient for rogue (*m* = 1) or clustered (*m* = 10) RyRs.

**REFERENCES**

Brochet, D.X.P., Xie, W.J., Yang, D.M., Cheng, H.P., and Lederer, W.J. (2011). Quarky calcium release in the heart. *Circulation Research* 108(2)**,** 210-218. doi: 10.1161/circresaha.110.231258.

Chen, X., Kang, J.H., Fu, C.J., and Tan, W.C. (2013). Modeling calcium wave based on anomalous subdiffusion of calcium sparks in cardiac myocytes. *Plos One* 8(3)**,** 9. doi: 10.1371/journal.pone.0057093.

Izu, L.T., Wier, W.G., and Balke, C.W. (2001). Evolution of cardiac calcium waves from stochastic calcium sparks. *Biophysical Journal* 80(1)**,** 103-120.

Li, J.H., Xie, W.J., Chen, X., Huo, Y.L., Cheng, H.P., and Tan, W.C. (2017). A novel stochastic reaction-diffusion model of ca2+ blink in cardiac myocytes. *Science Bulletin* 62(1)**,** 5-8. doi: 10.1016/j.scib.2016.12.001.

Metzler, R., and Klafter, J. (2000). The random walk's guide to anomalous diffusion: A fractional dynamics approach. *Physics Reports-Review Section of Physics Letters* 339(1)**,** 1-77. doi: 10.1016/s0370-1573(00)00070-3.

Smith, G.D., Keizer, J.E., Stern, M.D., Lederer, W.J., and Cheng, H.P. (1998). A simple numerical model of calcium spark formation and detection in cardiac myocytes. *Biophysical Journal* 75(1)**,** 15-32.

Sobie, E.A., Dilly, K.W., Cruz, J.D., Lederer, W.J., and Jafri, M.S. (2004). Termination of cardiac of ca2+ sparks: An investigative mathematical model of calcium-induced calcium release. *Biophysical Journal* 86(5)**,** 3329-3329. doi:10.1016/S0006-3495(02)75149-7

Walker, M.A., Williams, G.S.B., Kohl, T., Lehnart, S.E., Jafri, M.S., Greenstein, J.L., et al. (2014). Superresolution modeling of calcium release in the heart. *Biophysical Journal* 107(12)**,** 3009-3020. doi: 10.1016/j.bpj.2014.11.003.

Zahradnikova, A., Valent, I., and Zahradnik, I. (2010). Frequency and release flux of calcium sparks in rat cardiac myocytes: A relation to ryr gating. *Journal of General Physiology* 136(1)**,** 101-116. doi: 10.1085/jgp.200910380.
